# PKG Movement Recording System Use Shows Promise in Routine Clinical Care of Patients With Parkinson's Disease

**DOI:** 10.3389/fneur.2019.01027

**Published:** 2019-10-01

**Authors:** Rajeshree Joshi, Jeffrey M. Bronstein, A. Keener, Jaclyn Alcazar, Diane D. Yang, Maya Joshi, Neal Hermanowicz

**Affiliations:** ^1^Clinical Partners Group, Santa Monica, CA, United States; ^2^Department of Neurology, University of California, Los Angeles School of Medicine, Los Angeles, CA, United States; ^3^Department of Neurology, University of California, Irvine, Irvine, CA, United States

**Keywords:** neurology, movement disorder, Parkinson's disease, digital health, personal Kinetigraph, objective measurement

## Abstract

Parkinson's disease (PD) is a debilitating, neurodegenerative disorder that affects nearly one million people. It's hallmark signs and symptoms include slow movements, rigidity, tremor, and unstable posture. Additionally, non-motor symptoms such as sleeplessness, depression, cognitive impairment, impulse control behaviors (ICB) have been reported. Today, treatment regimens to modify disease progression do not exist and as such, treatment is focused on symptom relief. Additionally, physicians are challenged to base their diagnoses and treatment plans on unreliable self-reported symptoms, even when used in conjunction to validated assessments such as the Unified Parkinson's Disease Rating Scale (UPDRS) and clinical exams. Wearable technology may provide clinicians objective measures of motor problems to supplement current subjective methods. Global Kinetics Corporation (GKC) has developed a watch-device called the Personal KinetiGraph (PKG) that records movements and provides patients medication dosing reminders. A separate clinician-use report supplies longitudinal motor and event data. The PKG was FDA-cleared in September 2016. We studied 63 PD patients during 85 routine care visits in 2 US academic institutions, evaluating the clinical utility of the PKG. Patients wore a PKG for 6 continuous days before their visit. Next, PKG data was uploaded to produce a report. In clinic, physicians discussed PD symptoms with patients and conducted a motor examination prior to reviewing the PKG report and comparing it to their initial assessments. Lastly, patient, caregiver and physician satisfaction surveys were conducted by each user. Across all visits when patients did not report bradykinesia or dyskinesia, the PKG reported these symptoms (50 and 33% of the time, respectively). The PKG provided insights for treatment plans in 50 (79%) patients across 71 (84%) visits. Physicians found improved patient dialogue in 50 (59%) visits, improved ability to assess treatment impact in 32 (38%) visits, and improved motor assessment in 28 (33%) visits. Patients stated in 82% of responses that they agreed or strongly agreed in PKG training, usability, performance, and satisfaction. In 39% of responses, they also reported a very valuable impact on their care. PKG use in 63 PD patients within our clinical practice showed clinically relevant utility in many areas.

## Introduction

PD is a progressive, neurodegenerative disease causing motor and non-motor symptoms such as tremor, rigidity, slow movements, and postural instability (1). Non-motor symptoms may include sleep disturbances, bladder and bowel dysfunction, and cognitive impairment (2). To date, no intervention has shown efficacy or is designated as being useful in clinical practice as a means of preventing or slowing PD disease progression and as such treatment is focused on symptom management (3). Treatment regimens rely on assessments such as the UPDRS and clinical exams. Both include patient -reported symptoms which are often inaccurate or incomplete (4). currently affects approximately one million people in the United States with an anticipated large increase in the near future. The Parkinson's Foundation Prevalence Project estimates that 930,000 people in the United States will be living with PD by the year 2020. This number is predicted to rise to 1.2 million by 2030 (5). The main motor related symptoms of Parkinson's disease are bradykinesia, rigidity, tremor, and postural instability. Other potential manifestations are myriad and may include autonomic, behavioral, cognitive, olfactory, sensory, sleep, and visually related dysfunctions, now encapsulated in the moniker, “Non-Motor Manifestations of Parkinson disease” (1). Although PD has common, basic motor phenomena, the phenotype is highly variable.

Most of the current therapies for PD are directed toward reducing rigidity, improving bradykinesia and consequently improving mobility. Tremor, however, is poorly understood (6). Since the introduction of L-DOPA use in PD in the late 1960's by George Cotzias and collaborators, it has remained the gold-standard of medical therapy. Despite the robust and consistent benefits in early disease, advanced patients often experience motor fluctuations (due to the short drug half-life), L-DOPA-induced dyskinesias and varied absorption with dose failures (1). As time passes the interval of benefit from levodopa often shortens, resulting in so-called “wearing off,” and the response may be less predictable, with “off” episodes not clearly associated with medication timing. Levodopa-induced dyskinesias occur in up to 80% of patients with Parkinson's after a few years of chronic treatment (7). Impulse control behaviors (ICB) are recognized as non-motor complications of dopaminergic medications in patients with Parkinson's disease (8). Therefore, treatment of non-motor symptoms requires monitoring when medications are either adjusted or other therapies are introduced. The clinician's challenge becomes providing recommendations for medication regimens that address these problems without being unrealistically complicated or provocative of intolerable side effects. This is not a simple task. Treatment recommendations are further complicated by the clinician's dependence upon information provided by the patient and their care partner regarding the timing of symptoms, or correctly identifying symptoms, e.g., when mobility is poor, or discrimination between tremor and dyskinesia. Lastly, a well-known phenomenon among clinicians is that patients tend to do better during their appointments and their examination does not necessarily reflect their performance at home: “Why don't you walk like that when we are at home?” is an often-heard comment from spouses. A moment's reflection reveals what an archaic, inaccurate and frustrating process the current method of PD patient evaluation and treatment is for all concerned.

Formal clinical assessment tools such as the Unified Parkinson's Disease Rating System (UPDRS), and questionnaires such as Parkinson's Disease Questionnaire−39 (PDQ-39), 24-h motor diaries also have well-known limitations (9, 10).

Advanced cases requiring invasive PD therapies of intrajejunal infusion of carbidopa/levodopa (Duopa in the U.S., Duodopa elsewhere) and Deep Brain Stimulation (DBS) require thoughtful patient selection based upon levodopa responsiveness and well-characterized motor problems, including motor fluctuations or troublesome tremor (11).

In a systematic review of existing technologies Rovini et al. (12), included 147 articles in their analysis. They abstracted data from the papers and considered: the used technological solutions and typology of sensors, their placement over the body and the sampling frequency; the experimental protocol adopted; the subjects involved, according to their pathology and their health status; the performed analysis, including the extracted features, the applied statistical methods, the implemented classifiers and the main findings for each work. Particular attention was focused on the classifiers performance because they can synthetically represent the robustness of the technology proposed for a specific PD application. They identified several key characteristics of reliable and accurate wearable sensors and algorithms: a 3-axis accelerometer and power spectral density for signal interpretation.

**Figure 1:**
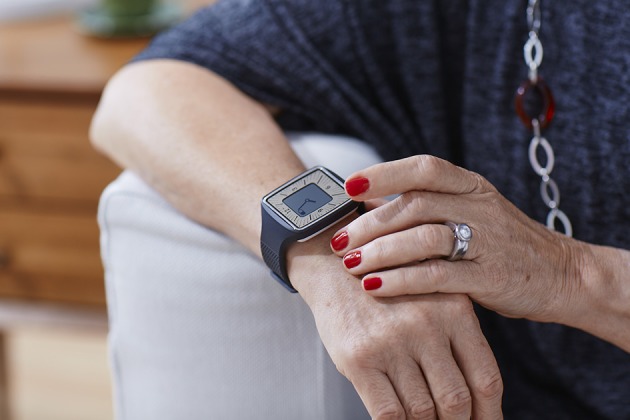
PKG watch.

**Figure 2:**
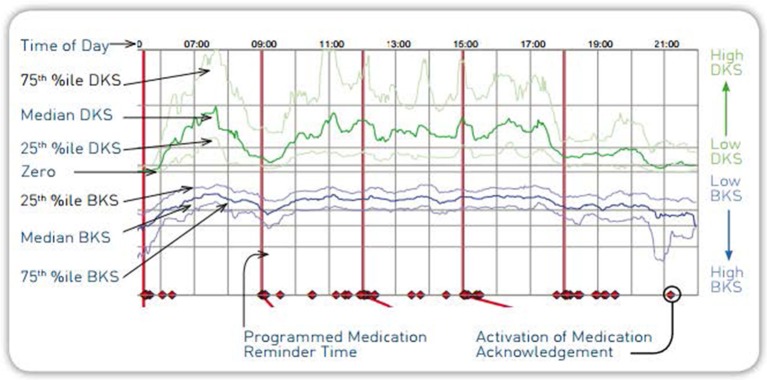
PKG report.

The Personal KinetiGraph (PKG) System was developed by neurologists at the Melbourne-based Florey Institute of Neuroscience and Mental Health in response to the lack of objective measurement tools for movement disorders symptoms. It is intended to quantify kinematics of movement disorder symptoms in conditions such as PD, including tremor, bradykinesia, and dyskinesia. It includes a medication reminder, medication administration marker, and is intended to monitor activity associated with movement during sleep. The system is indicated for use in individuals 46–83 years of age and was FDA-cleared in September 2016 (13). The PKG System consists of the PKG watch (Figure 1) and PKG Report (Figure 2).

The PKG watch is a wrist-worn medical device that looks like a wristwatch. It is programmed and dispensed by clinic staff and worn by the patient for 6–10 full days. During this wear time, the PKG watch automatically collects data on the type of movement experienced by the patient and reminds the patient to register when they have taken dopaminergic medications as prescribed by their physician. A proprietary mathematical algorithm translates the raw movement data collected by the PKG watch into a PKG report. It is intended that the physician use the PKG as an additional tool for clinical care. We investigated this system to assess its clinical utility in capturing motor problems within the patient's home setting, in improving dialogue with the patient, and its impact on treatment recommendations.

The PKG has been studied by various investigators. It is our intent to build upon these earlier research efforts.

## Methods and Materials

### Study Design

This was a single-arm, open-labeled, investigator-sponsored, observational study conducted at two US academic institutions: University of California, Irvine (UCI) and University of California, Los Angeles (UCLA). Each investigational site's local University Institutional Review Board reviewed and approved the study protocol prior to subject enrollment.

### Objectives

The primary purpose of this study was to evaluate the clinical utility of the PKG Movement Recording System when used in routine clinical care of patients with PD. Secondary objectives included PKG performance and user satisfaction.

### Study Population

Active patient charts within each practice were used to identify potential subjects. Male and female patients with PD, between 46 and 83 years of age at the time of consent, responsive to dopaminergic therapy and were walking without routine use of a walking-aid were included. Patients with psychosis were excluded from study participation. Subjects were consented either in person or by phone. Each subject could take home, review, and ask questions regarding the Informed Consent Form and study.

During the study subjects were treated according to each clinician's standard practice and for no <90 days.

### Endpoints

The primary endpoint was the frequency of disagreement of PD symptoms reported by the patient vs. the PKG Report.

#### Secondary Endpoints Were:

1. Frequency of treatable findings per clinician's assessment of PKG report
2. Frequency of PKG report use in treatment change decisions
3. Global impression of improvement
4. Global impact on patient care
5. PKG subject and caregiver satisfaction
6. PKG Scores: Fluctuation Score, Bradykinesia Score, Dyskinesia Score.

### Device Description

The PKG System was developed in response to the lack of objective measurement tools for movement disorders symptoms. It is intended to quantify kinematics of movement disorder symptoms in conditions such as PD, including tremor, bradykinesia, and dyskinesia. Its functionality measures acceleration of the wrist and includes a medication reminder, medication administration marker. It is intended to monitor activity associated with movement, including movement during sleep. The system is indicated for use in individuals 46–83 years of age and was FDA-cleared in September 2016. The PKG System consists of the PKG watch (Figure 1) and PKG Report (Figure 2).

The PKG is worn like a wrist watch (on the most affected side), weighs 35 g, and contains a rechargeable battery and a 3-axis iMEMS accelerometer (ADXL345 Analog Devices) set to record 11-bit digital measurement of acceleration with a range of ±4 g and sampling rate of 50 samples per second using a digital micro-controller and data storage on flash memory. It is programmed and dispensed by clinic staff and worn by the patient for 6–10 full days. During this wear time the PKG watch automatically collects data on the type of movement experienced by the patient and reminds the patient to register when they have taken dopaminergic medications as prescribed by their physician. The acceleration recordings were divided into 2-min epoch and the inputs to the expert system included Mean Spectral Power (MSP) within bands of acceleration between 0.2 and 4 Hz, peak acceleration and the amount of time within these epochs that there was no movement. The algorithm similarly recognizes dyskinesia as having movements of normal amplitude and acceleration but with shorter periods without movement. Off periods are correlated with dose administration. Off periods are seen as bradykinesia half an hour prior to medication administration, but differentiated from true bradykinesia when dyskinesias appear 1 h afterward medication administration. This proprietary mathematical algorithm that was developed using a fuzzy logic approach, the spectral power of the low frequencies of an accelerometer trace and with iterative modeling and retesting of new subjects translates the raw movement data collected by the PKG watch into bradykinesia and dyskinesia scores and a graphical PKG report (14).

### Questionnaires

Two surveys to help assess clinical utility were administered during the course of the study: (1) *Global Impact on Patient Care* and (2) *PKG Patient/Caregiver Survey:*

#### Global Impact on Patient Care–

This survey was completed by physicians at the end of a patient's study participation in order to determine if and how the PKG system impacted various aspects of the patient's clinical care, including patient education. It consisted of two subparts: *Global Impact on Patient Care* (addressing overall impact on patient care) and *Clinical Global Impression of Improvement* (overall impression on patient improvement). Examples of questions asked of physicians included:

- Improved dialogue with patient?
- Improved patient education about illness?
- Improved patient education about symptoms?
- Improved ability to assess impact of a therapy?
- Improved ability to assess patient PD symptoms?

The complete set of questions and results for subpart *Global Impact on Patient Care* can be found in **Table 5**. Subpart *Clinical Global Impression of Improvement* could not be completed since the majority of subjects did not require a follow-up visit per their treatment plan.

#### PKG Patient/Caregiver Survey

Subjects and their caregivers were asked to complete a three-part questionnaire to address (1) *PKG Use* (product usability), (2) the *PKG Impact on Patient Care*, and (3) *Patient Global Impression* (patient overall impression). Examples of questions asked subjects and their caregivers included:

- Was the training received on the use of the PKG adequate?
- Was the PKG easy to use?
- Did PKG medication reminders assist you in taking medication on time?
- Did PKG data assist you with explaining your symptoms to your doctor?

The complete set of questions and results for subparts PKG Use *Global Impact on Patient Care* and *PKG Impact on Patient Care* can be found in **Tables 6**, **7**, respectively. Subpart *Global Impression* was not analyzed due to a low response rate.

### PKG Report

The PKG report is a graphical illustration of the patient's movement as recorded by the PKG during their 6-day wear period (Figure 2). The PKG report provides a measure of severity and proportion of time spent at various levels of dyskinesia and bradykinesia in relation to timing of levodopa medication. The main plot of the PKG report shows the median, 25 and 75% percentile of the bradykinesia score (BKS) and dyskinesia score (DKS) of the patient over all days of recording compared with the BKS and DKS of a control group (non-PD patients). In addition, a separate tremor assessment provides tremors relative to medication administration to illustrate “off periods”^1^.

### Procedures

All subjects were assessed according to each clinician's standard practice, approximately every 3 months. For 6 consecutive days prior to a visit, patients wore a PKG watch which collected movement data and provided medication reminders. Watches were removed only for showering or bathing. Data from the watches were subsequently uploaded in the clinic to generate a PKG report. Patients who were enrolled in the study underwent a screening examination. In each baseline and follow-up visit, the physician followed a protocol of first assessing and interviewing the patient while blinded to PKG data and noting treatment adjustments. Then the PKG report was unblinded to both the physician and patient. Upon review of the PKG data, the physician reassessed the patient, and adjusted the treatment plan in light of new information. Physicians recorded the change in the treatment and plan including whether or not the decision was based upon additional data that the PKG provided them.

Data from the PKG report was used in both primary and secondary endpoint analyses. Safety was also assessed at each visit. Medications were reviewed prior to enrollment and at each visit to confirm responsiveness to dopaminergic treatment and to adjust per revised treatment plans. Lastly, questionnaires were administered to measure physician, patient and caregiver PKG satisfaction rates.

### PKG Scores: Fluctuation and Dyskinesia Score (FDS), Bradykinesia Score (BKS), Dyskinesia Score (DKS)

The proposed optimal and acceptable PKG Score targets used in this study were based on published reports, where the FDS is a summary score of motor fluctuation and dyskinesia, derived as the logarithm of the sum of the interquartile range of BKS and DKS across all days of recording and has been shown to differentiate clinical fluctuators from non-fluctuators (10).

Bradykinesia (BKS)—Optimal: median BKS score < 23 and FDS > 8; acceptable: median scores between 23 and 25 and/or Fluctuations (FDS) > 7.5 with no fluctuations; Dyskinesia (DKS)—Optimal: median DKS score < 7 and FDS < 10.8; acceptable: median scores between 7 and 9 and FDS < 13 and no fluctuations.

### Statistical Analysis

Descriptive statistics including range, median, absolute values, proportion of visits, and proportion of patients were used in all analyses. A per-protocol analysis was conducted on the primary analysis set. Data for both primary and secondary analyses were presented as both proportion of visits and proportion of patients.

## Results

Overall, information from the PKG report augmented the patient-physician dialogue about symptoms and the physician treatment plan decisions.

Subject Demographics: this study enrolled 63 adult patients with Parkinson's disease. Baseline characteristics of the study population include a median age of subjects at 65 years and slightly more male enrollees than females (59% vs. 41%, respectively) (Table 1).

**Table 1:** Subject demographics & Medication at enrollment.

	**Overall**		**Site 01**		**Site 02**	
*n* (%)	63	100%	42	67%	21	33%
Age						
Median	68.5		68		70	
(Min, Max)	43	86	54	86	43	80
Gender, *n* (%)						
Female	26	41%	18	43%	8	38%
Male	37	59%	25	60%	12	57%
Average Duration of PD (Year)	10.8		11.8		8.8	
			**Overall**			
**Medication at enrollment by drug class**			***n***		**%**	
Amantadine			15		24	
COMT-inhibitor			10		16	
Dopamine agonist			32		51	
Decarboxylase inhibitor			1		2	
Duopa			1		2	
Levodopa			61		97	
MAOB-inhibitor			13		21	
Other			5		8	

### Number of Study Visits:

Study visits were scheduled according to routine care. In total, 85 visits' data was used for primary and secondary analyses:

- 65 screening and enrollment visits
- 54 baseline visits
- 16 follow up #1 visits
- 8 follow up #2 visits
- 5 follow up #3 visits
- 2 follow up #4 visits

### Primary Analysis

Our primary endpoint was the frequency of disagreement of symptoms reported by the patient vs. PKG report (i.e., the number of times a symptom was present in the PKG report and not reported by the subject when interviewed by the investigator, or was reported by the subject but not present in the PKG report). Across all 85 visits, symptoms were identified by the PKG report and not reported by the subject 30 times (35%). In contrast, the subject reported a symptom that did not appear in the PKG report 15 times (17.6%). Table 2a provides the distribution of symptoms, while Table 2b provides the proportion of patients who experienced disagreement in symptom reporting. Bradykinesia represented 50% of the symptoms reported by the PKG report and not by the subject. Proportionately, 48% of patients had a PKG reporting a symptom that they had not told their physician about and 24% of patients reported a symptom that hadn't appeared in the PKG report.

**Table 2A:** Frequency of disagreement in Symptom reporting between subject and PKG report across visits.

	**Symptoms reported by PKG report AND NOT reported by subject**		**Symptoms reported by subject AND NOT reported by PKG report**	
**Symptoms**	**(*n*)**	**(%)**	**(*n*)**	**(%)**
Bradykinesia	15	50	6	40
Dyskinesia	10	33	6	40
Tremor	5	17	3	20
Total	30	35	15	17.6

**Table 2B:** Proportion of patients who experienced disagreement in symptom reporting between subject and PKG report.

	**Symptoms reported by PKG report AND NOT reported by subject**		**Symptoms reported by subject AND NOT reported by PKG report**	
**Symptoms**	**(*n*)**	**(%)**	**(*n*)**	**(%)**
Bradykinesia	15	24	6	10
Dyskinesia	10	16	6	10
Tremor	5	8	3	5
Total	30	48	15	24

### Secondary Analyses

#### Frequency of Treatable Findings Identified in PKG Report

We measured frequency of treatable findings, and impact on treatment plans and patient care. Table 3 shows all events in which the PKG reported a treatable finding to the clinician for potential action in clinical practice. Bradykinesia represented 61% of all treatable findings, followed by dyskinesia as 32% of all treatable findings. Global bradykinesia requiring one or more doses of levodopa or other treatment to be increased was the most frequently observed subtype in 47% of bradykinesia cases, predictable dyskinesia requiring one or more treatments represented 44% of dyskinesia cases. In contrast, global dyskinesia requiring a reduction in one or more doses of levodopa, identification of impulsive-compulsive behaviors (ICB), nighttime sleep disturbances, and non-adherence to medication were least observed (0% of visits).

**Table 3:** PKG-identified treatable findings of symptoms and subtypes.

	**Total**	
	**(*n*)**	**(%)**
**Bradykinesia**	17	27
Global bradykinesia requiring one or more doses of levodopa or other treatment to be increased	8	13
Predictable bradykinesia with one or more doses (i.e., dose related fluctuations) requiring treatment	4	6
Unpredictable bradykinesia (e.g., off periods) with one or more doses and/or delayed response to some/all doses)	2	3
Early morning bradykinesia (i.e., bradykinesia prior to first dose of day)	3	5
**Dyskinesia**	9	14
Global dyskinesia requiring one or more doses of levodopa or other treatment to be reduced	0	0
Predictable dyskinesia with one or more doses (i.e., peak dose dyskinesia) requiring treatment	4	6
Unpredictable dyskinesia (off periods) with one or more doses	2	3
Type of dyskinesia (e.g., biphasic vs. peak)	3	5

#### Frequency and Percentage of PKG Report Use in Treatment Change Decisions

Data from the PKG reports was used to make 74 changes in treatment plans across 71 (84%) visits and 50 (79%) patients. Most common treatment changes included the addition of at least one medication or changed dosage and timing of medications.

Levodopa equivalent doses (LED) were calculated to determine how the PKG helped influenced treatment decisions at Enrollment and End-of-Study (EOS). Overall, an increase of 7.9 g of LED were prescribed (average of 125 mg/patient). Most often, however, treatment frequency or dose times were changed.

The PKG scores across all visits are provided in Table 4. Although the objective of this study was not to treat toward any pre-specified target, our secondary analyses included a PKG score measurement. Per Pahwa's et al. (10) assessment, our study population was managed optimally at EOS with Median BKS score < 23 (22.8), Median DKS score < 7 (2.3) and Median FDS > 8 (8.3).

**Table 4:** Study PKG scores.

	**PKG scores**			
	**Fluctuation score (FDS) (Optimal management FDS > 8)**	**Bradykinesia score (BKS) (Optimal management BKS < 23)**	**Dyskinesia score (DKS) (Optimal management FDS < 7)**	**Percent time immobile (PTI)**
Median	8.3	22.8	2.3	6.1
Variance	11.0	37.0	14.5	34.7
SD	3.3	6.1	3.8	5.9
Min	3.7	11.7	0.1	0.4
Max	20.9	39.9	21.9	32.8

#### Global Impact of PKG on Patient Care

We also sought investigator's feedback on the impact of the PKG on patient care (Table 5). The investigators surveyed reported that the PKG improved their dialogue with the patient in 50 visits (59%), and improved education about their illness, symptoms, and ability to assess patient PD symptoms in 65 visits (77%) (15, 29, and 33%, respectively).

**Table 5:** Global impact of PKG on patient care.

	**Count**	**Visits (%)**
Improved dialogue with patient	50	59
Improved patient education about illness	13	15
Improved patient education about symptoms	25	29
Improved patient education about treatment use	7	8
Improved ability to assess impact of a therapy	32	38
Improved ability to assess need for additional tests or treatments	6	7
Improved ability to assess patient PD symptoms	28	33
Avoidance/cancellation of a referral that was previously planned	1	1
Patient referred for advanced therapy evaluation now became optimally medically managed (avoided advance therapy)	0	0
Other	2	2

#### PKG Subject and Caregiver Satisfaction

Subjects and caregivers were surveyed to better understand their satisfaction levels with the PKG (Table 6). Of the responses, 82% agreed or strongly agreed that the PKG was easy to learn, was easy to use, enabled them to confirm medication administration, performed as expected and would use it again.

**Table 6:** Patient/Caregiver survey - PKG use.

	**Strongly disagree**	**Disagree**	**Neutral**	**Agree**	**Strongly agree**	**No response**
The training I received on the use of the PKG was adequate	2	1	2	26	44	10
The PKG was easy to use	2	1	0	25	47	9
The PKG performed as I expected	2	4	2	28	34	9
I was able to wear the PKG and complete medication use confirmations as instructed by my doctor	1	3	0	27	43	9
I would be willing to use the PKG again to assist in the management of my PD in the future	1	0	1	34	39	9
Count	8	9	5	140	207	46
Overall responses (%)	2	2	1	33	49	11

#### PKG Impact on My Care

In addition, subjects were surveyed to assess the PKG's impact on their care (Table 7). Across all questions and responses, 74% of all responses reported the PKG to be somewhat or very valuable in helping patients take medication on time, explain symptoms, provide additional data to their manage care for PD. Providing additional data about patient movement during normal daily activities was the most favorable response.

**Table 7:** PKG impact on my care.

	**Not valuable**	**Of little value**	**Neutral**	**Somewhat valuable**	**Very valuable**	**No response**
PKG medication reminders assisted me with taking my medication on time	1	1	10	30	34	9
PKG data assisted me with explaining my symptoms to my doctor	1	3	20	27	24	8
The PKG provided data to my doctor about my symptoms that I could not provide	1	2	13	26	34	8
The PKG provided additional data about my movement during normal daily activities	0	1	4	32	38	8
The PKG provided additional data that assisted my doctor with making decisions about my care	0	1	9	30	36	8
The PKG provided additional data that contributed to the overall management of my PD	1	0	9	31	35	8
Count	4	8	65	176	201	49
Responses (%)	1	2	13	35	39	10

#### Case Studies

One example of a patient who benefited from PKG use is a 75-year-old female with a 7-year history of PD. She has mild cognitive impairment (MCI) and complained of motor fluctuations. Due to her MCI, she had difficulty reporting when she was off and when she was dyskinetic. The PKG identified off times in the AM and dyskinesias in the afternoon. Based on the PKG data her regimen of carbidopa/levodopa dose was adjusted and her symptoms markedly improved.

Another example of a patient who benefited from PKG across multiple visits is a 58-year-old female with a 14-year history of PD. The patient reported bradykinesia but was reluctant to make a treatment change. After review of the PKG findings with the patient she chose to adjust her treatment regimen. A second PKG report at a follow-up visit characterized dyskinesias which were then addressed by another treatment adjustment. The patient was ultimately placed on enteral carbidopa/levodopa. The clinician felt that the PKG report improved dialogue with the patient, thus improving patient education about her illness and symptoms, allowing her to make better informed decisions about her care. The initial Patient Caregiver survey response agreed regarding adequate training, ease of use, performance, medication administration, and willingness to use again. At the end of the study, the response was “Strongly Agreed” when asked the same questions. Furthermore, the patient responded the PKG system had a very valuable impact on her care.

#### Safety

Safety was assessed at each visit. No serious adverse events or adverse device effects were reported during the course of this study.

## Discussion

Treatment of PD patients is heavily reliant upon patient and caregiver reported symptoms. Standard assessments such as the UPDRS incorporate patient-reported symptoms to monitor and determine disease manifestations and progression. This may be compromised by the cognitive impact of PD, the misidentification of symptoms (e.g., tremor vs. dyskinesia), and the variability of symptoms. As a result, symptoms may be misreported or underreported. With the recent proliferation of wearable devices in healthcare, we were interested to see if the PKG could be used in clinical practice to provide real-world data through passive data collection and medication reminders. We were particularly interested to see how the data might identify symptoms that the patients had not reported, altering patient-physician dialogue, if these data could provide an objective basis to aid treatment decisions. Also, importantly, we wished to assess the patient perspective on the use of the wearable device in the management of their symptoms.

Using objective measurement (OM) to augment PD treatment has been an area of interest for quite some time. Rovini et al. (12) analyzed several wearable technologies in 2017 to offer guidance on reliable features and methods. Their review focused on wearable devices for PD applications and identified five main fields: early diagnosis, tremor, body motion analysis, motor fluctuations (ON–OFF phases), and home and long-term monitoring. Their aim was to capture observations throughout disease progression, e.g., early symptoms upon diagnosis, during disease progression some of the most complex conditions (i.e., motor fluctuations and long-term remote monitoring).

Pahwa et al. (10) published an expert review in 2018 to provide additional recommendations on the role of the PKG in routine clinical assessments. A group of 5 neurologists focused on the use of the PKG as a tool to inform clinical assessment and impact patient care. The exact clinical scenarios as documented in the paper were:

- The poor historian: patient is unable to identify or communicate PD symptoms or their report of the symptoms does not match the clinical impression;
- Motor fluctuations: patient presents with motor fluctuation symptoms of complex or uncertain pattern and severity, and/or increased OFF-time;
- Dyskinesia: patient presents with dyskinesias of uncertain pattern or severity, clinical impression of dyskinesia does not match patient report, and/or movements of uncertain etiology;
- Tremor: patient over/under reports tremor, or denies any tremor response to medication;
- Dyskinesia vs. Tremor: patient reports involuntary movement of unclear type, with inability to distinguish between tremor and dyskinesias over the day, or how they are affected by medications;
- Excessive daytime sleepiness: over/under reporting of daytime sleepiness by patient, or incongruences between patient/caregiver reports;
- Response to therapy change: patient is unclear about the benefits of a therapeutic change;
- Assessment for and optimization of advanced therapies: assessing the need for advanced therapies such as DBS in patients with suspected motor fluctuations, dyskinesias, and/or tremor. Advanced therapies also provide more complexity, making optimization more challenging;
- Telemedicine: patients who cannot be seen in the clinic in person, leading to more limited telemedicine-based assessments;
- New patients: any newly diagnosed PD patient or a new patient to the clinic (10).

They concluded the PKG may augment clinical decision-making. For example, objective measurement helps detect uncontrolled patients and provide data to help decide on which treatments should be introduced or changed to optimize therapy (10). Ultimately, they stated that the objective measures of the PKG could confirm presence/absence of motor manifestations (including fluctuations) as reported by the patient, provide discussion points for physician/patient dialogue, provide interim data between clinic visits, and finally can be used to establish a baseline for newly diagnosed patients (10).

In 2018, Farzanehfar et al. (15) reported successful use of the PKG in routine PD to improve outcomes by adjusting therapy to achieve predefined target ranges of OM. In this study, an accelerometry based measurement and predefined target ranges were used to assess motor function in a Northern Tasmania PD cohort managed by a Movement Disorder clinic. Approximately 40% (*n* = 103) of the total PD patients within the clinic participated in this study. Targets were set to differentiate between controlled and uncontrolled bradykinesia and dyskinesia. Farzanehfar's team used targets and treatment guidelines created by a panel of four neurologists and scientists who were familiar with PKG use including M. Horne and K. Kotschet. Targets as directly stated in the paper were: BKS = 23 corresponds to a UPDRS III of ~20, and BKS = 25 to a UPDRS III of ~30. Bradykinesia was considered controlled if the BKS < 23 and uncontrolled if the BKS > 26. Changes in therapy were not required if the BKS < 23. For Dyskinesia targets (where DKS represents the median dyskinesia score for the PKG recording period). Control was defined as a DKS < 9, which corresponds to an Abnormal Involuntary Movement Score (AIMS) of 10. DKS that peaked between 7 and 9 with an FDS > 13 were treated (15).

Earlier this year, Santiago et al. (16) reported the use of the PKG in routine care of PD patients led to medication adjustments in approximately one third of 112 records. Physicians identified patients in routine care for whom an objective measurement could improve physician-patient interactions during clinic visits. Patients wore a PKG for ≥6 days during routine daily living activities. The survey period ran between December 2015 through July 2016 and four physicians provided survey responses. Of 112 completed physician surveys, 46 (41%) indicated the PKG provided relevant additional information sufficient to consider adjusting their therapeutic management plan; 66 (59%) indicated the PKG provided no further information to support a therapeutic decision differing from that made during a routine clinical evaluation. Upon further review of these 46 surveys, 36 surveys (78%) revealed the information provided by the PKG ultimately resulted in adjusting the patient's medical management (16).

We were interested in building upon these efforts by understanding the clinical utility in practice from both the physician and patient perspectives.

The average duration of PD in our study population was over 11 years. The patients were managed by experienced movement disorders clinicians, and yet, with the PKG system, a 35% improvement in motor-symptom monitoring was accomplished. The PKG measures movement every 2 min. The algorithm utilizes medication administration event data and prolonged immobility to distinguish between intentional immobility, e.g., sleep, vs. bradykinesia. It bases this calculation upon a percent-time-immobile score that is then cross-checked against patient responses (17). Furthermore, we felt understanding fluctuations more accurately through empirical information would help enhance patient dialogue and treatment planning. The use of the PKG Fluctuation and Dyskinesia Score based upon a difference in the interquartile ranges of patient movement was selected as a secondary objective (18).

The clinical utility of the PKG system is supported by both the positive patient reports of using the PKG, and the clinicians reporting improved patient dialogue and educational opportunity.

Future research opportunities for the PKG system in Parkinson's disease include:

- PKG as a replacement for patient diaries in clinical trials assessing on-time, off-time, quality of on-time, and dyskinesias;
- Further assessment of PKG measures in correlation with scales for patient quality of life, sleep issues, both at night and daytime sleepiness, impulse control disorder, and patient and clinician impression of improvement.

Study limitations included a small sample size of patients, per-protocol analysis, open label design, and no evaluation of motor function after PKG-informed changes in therapy. Additionally, we did not achieve our secondary endpoints of Clinical Global Impression of Improvement (CGI-I) and Patient Global Impression of Improvement (PGI-I) as only 16 patients completed a follow up visit beyond baseline. Also, the PKG system has limitations due to its ability to measure directly only some of the motor manifestations of Parkinson's disease, which for many patients do not comprise the most troubling symptoms. The PKG does measure overall bradykinesia, tremor and dyskinesia but only on one side and does not directly evaluate gait, freezing or falls. It remains to be determined with further experience what non-motor symptoms may be indirectly inferred from the PKG recordings or if it could be useful in correlating motor symptoms with MCI subtypes (19). This in, turn may help physicians determine how to best integrate PKG data with patient symptom reporting and medical management (15) or if objective measurement in routine care could improve outcomes (16).

The PKG system provided clinical utility through improved characterization of motor manifestations of Parkinson's disease, both the type and timing. This served to improve physician-patient dialogue and provide insight to clinicians and patients to inform treatment impact and decisions. Additionally, patients who wore the PKG watch felt the medication administration reminders were valuable. Patients indicated the watch was easy to use, performed as expected, and they would wear the PKG again. Furthermore, patients were satisfied with PKG training, usability and performance. Many patients found that the PKG had a valuable impact on their care.

For optimal care of the PD patient, moving from sole reliance upon subjective symptom reporting with office based clinical examinations separated by weeks or months to incorporation of objective measurements by wearable devices is intuitively reasonable and increasingly supported by several studies. In our experience and that of others use of the PKG system has demonstrated value in care of the PD patient.

## Data Availability Statement

The datasets generated for this study are available on request to the corresponding author.

## Ethics Statement

This study was carried out in accordance with the Laws of California, UC Regents, UCI, UCLA, policies, GCP, and HIPAA guidelines with written informed consent from all subjects. All subjects gave written informed consent in accordance with the Declaration of Helsinki. The protocol was approved by UCI Office of Research Institutional Review Board and UCLA Office of Human Protection Research Program Institutional Review Board.

## Author Contributions

NH was lead PI and author on study and responsible for overall study design, protocol development, ICF development, CRF design, development of physician, patient and caregiver questionnaires, patient identification, recruitment, consent, clinical management of patients, study execution, site data review, case study provision, and overall study oversight at UCI and study-wide. RJ was responsible for managing overall study timelines, data monitoring and analysis, supporting overall study scientific affairs, and medical writing of the manuscript. JB was co-PI on the study and responsible for study design, study protocol development, ICF development, CRF design, development of physician, patient and caregiver questionnaires, patient identification, recruitment, consent, clinical management of patients, study execution, site data review, case study provision, and overall study oversight at UCLA. AK was co-PI on the study and responsible for study design, study protocol development, ICF development, CRF design, recruitment, consent, clinical management of patients and study execution. JA and DY was responsible for study coordination, patient recruitment, consent, data entry and site data review at UCI. MJ was responsible for data review and analysis of all physician, patient, and caregiver surveys for overall study.

### Conflict of Interest

The authors declare that this investigator-initiated study received funding in the form of a restricted grant from Global Kinetics Corporation, Ltd. The funder had the following involvement with the study: device provision, software to generate PKG reports, and technical support for device malfunction. All authors declare no conflict of interest.

## Footnotes

^1^www.globalkineticscorporation.com/for-healthcare-professionals/
